# Genetic testing and research in Lynch Syndrome - is it a choice or a responsibility?

**DOI:** 10.1186/1897-4287-9-S1-P6

**Published:** 2011-03-10

**Authors:** Lorraine Cowley, Janice McLaughlin, Tracy Finch, Emma Clavering, John Burn

**Affiliations:** 1 Institute of Human Genetics, Newcastle University, International Centre for Life, Central Parkway, Newcastle upon Tyne, NE1 3BZ, UK; 2 PEALS (Policy, Ethics and Life Sciences Research Centre), Newcastle University Citywall, Citygate, St. James’ Boulevard, Newcastle upon Tyne, NE1 4JH, UK; 3 Institute of Health and Society, Newcastle University, Baddiley-Clark Building, Richardson Road, Newcastle upon Tyne, NE2 4AX, UK

## Background

This project explores experiences of an extended family; one of the first internationally to have the MLH 1 gene characterised [1], causing what has become known as Lynch Syndrome. The paper focuses on how participants frame notions of choice and responsibility in the context of genetic testing and research.

## Method

A sample of 15 from 50 of the biomedical family who tested either positive or negative for MLH1 was invited to discuss family relationships. The methodology used multiple qualitative interviews and visual methods including photo elicitation, social mapping and engagement with the genetic family pedigree. Data was analysed from a narrative perspective [2].

## Results

Choice in genetic testing and research does not appear to be the key value for participants in this study; instead they are influenced by a sense of responsibility [3]. Choice for them is an important right, one exercised by other family members who declined a test. Their narratives however, illustrate three moral imperatives that transform choice into responsibility; they are: responsibility to children, to self and to scientific progress (the greater good).

Participants who were parents discussed a paramount duty of care to children as the main motivating factor when accepting a test. Those whose parents had declined genetic testing experienced disappointment and referred to the right to choose as a means of ethically managing that.

Genetic testing was also viewed in the context of a Health Belief model [4,5] framing those who declined testing as neglectful of a moral imperative to self care. Participants sometimes conflated choosing a genetic test with choosing life and used cautionary tales of those who declined testing and developed cancer to justify decisions and persuade others considering a test.

Within this moral framework an obligation to participate in genetic research is narrated from differing perspectives. A pioneering identity from being the first known family characterising the gene gave participants value and led to kin-like reciprocation invoking a desire to repay perceived medical investment in the “family”. A strong belief was held that genetics is key to the advancement of medicine.

## Conclusion

Genetic testing and participation in research is viewed as both a choice and a responsibility. The apparent conflict between rights to autonomy and moral imperatives of responsibility operate as caveats for preferencing or defending choice over responsibility or responsibility over choice. These insights contribute to important narratives about cultural engagement with the new genetics and have implications for practice.
